# Quantifying selective elbow movements during an exergame in children with neurological disorders: a pilot study

**DOI:** 10.1186/s12984-016-0200-3

**Published:** 2016-10-21

**Authors:** Hubertus J. A. van Hedel, Nadine Häfliger, Corinna N. Gerber

**Affiliations:** 1 Rehabilitation Center Affoltern am Albis, University Children’s Hospital Zurich, Mühlebergstrasse 104, CH-8910 Affoltern am Albis, Switzerland; 2 Children’s Research Center, University Children’s Hospital Zurich, Zurich, Switzerland; 3 Department of Health Sciences and Technology, ETH Zurich, Zurich, Switzerland

**Keywords:** Volitional motor control, Recovery, Compensation, Cerebral palsy, Exergame, Rehabilitation, Psychometrics, Validity

## Abstract

**Background:**

It is difficult to distinguish between restorative and compensatory mechanisms underlying (pediatric) neurorehabilitation, as objective measures assessing selective voluntary motor control (SVMC) are scarce.

**Methods:**

We aimed to quantify SVMC of elbow movements in children with brain lesions. Children played an airplane game with the glove-based YouGrabber system. Participants were instructed to steer an airplane on a screen through a cloud-free path by correctly applying bilateral elbow flexion and extension movements. Game performance measures were (i) % time on the correct path and (ii) similarity between the ideal flight path and the actually flown path. SVMC was quantified by calculating a correlation coefficient between the derivative of the ideal path and elbow movements. A therapist scored whether the child had used compensatory movements.

**Results:**

Thirty-three children with brain lesions (11 girls; 12.6 ± 3.6 years) participated. Clinical motor and cognitive scores correlated moderately with SVMC (0.50–0.74). Receiver Operating Characteristics analyses showed that SVMC could differentiate well and better than clinical and game performance measures between compensatory and physiological movements.

**Conclusions:**

We conclude that a simple measure assessed while playing a game appears promising in quantifying SVMC. We propose how to improve the methodology, and how this approach can be easily extended to other joints.

**Electronic supplementary material:**

The online version of this article (doi:10.1186/s12984-016-0200-3) contains supplementary material, which is available to authorized users.

## Background

In neurorehabilitation, one major physiotherapeutic goal is to restore the patients’ motor control as best as possible to maximize participation and quality of life of the patient. In recent years, the discussion has come up as to whether improvements should be induced by interventions exploiting restorative or compensatory strategies [1–3]. In a previous paper, Levin et al. [2] proposed how to define motor recovery and compensation at various domains according to the International Classification of Functioning, Disability, and Health (ICF) [4]. While we agree with their suggestions on the body structure domain (“Restoring function in neural tissue that was initially lost after injury” versus “Neural tissue acquires a function that it did not have prior to injury”) and body function domain (“Restoring the ability to perform a movement in the same manner as it was performed before injury” versus “Performing an old movement in a new manner”), we think it is not useful to define recovery or compensation at the activity level, as sufficient detail about the underlying movement physiology is lacking. As a consequence, in our opinion, measures that should be able to distinguish between changes in motor outcome following compensation or recovery should measure on the body structure or function domain.

Most currently applied clinical outcome measures cannot differentiate between improvements attained by compensatory or restorative approaches. We suggest that the quantification of selective voluntary movement control (SVMC) could be an appropriate outcome measure to assess changes in motor function and differentiate between restorative versus compensatory improvements. SVMC has been defined by an expert consensus group as the “ability to isolate the activation of muscles in a selected pattern in response to demands of a voluntary movement or posture” [5]. As timing, force, and speed of voluntary movements are controlled through the corticospinal tract, damage to this tract causes a loss of SVMC (e.g. Porter and Lemon [6]). Other upper motor neuron signs such as hypertonia, hyperreflexia or muscle weakness can additionally influence SVMC that is a commonly impaired motor function in patients with neurological diagnoses, including children with congenital or acquired brain lesions [7]. The inability to activate muscles in a physiological pattern in response to task-specific demands hampers motor function and affects motor development in children. A practical clinical assessment of SVMC would be valuable for clinical evaluation and decision-making.

In recent years, various robot-assisted and computer-supported systems have entered the field of pediatric rehabilitation [8]. Besides the general arguments that these systems might induce high numbers of repetitions and train physiological rather than compensatory movements, they should be advantageous in assessing (changes in) motor outcome. However, for example, Sivan et al. noted in their systematic review on outcome measures used in robot-supported trials for the upper extremity in adult patients after a stroke that only 3 out of 28 included papers included outcomes derived from the robotic systems [9]. Apparently, such advantages in assessments are currently not widely used.

In this study, we explored a playful method to assess SVMC in children with congenital and acquired brain injury. As children and adolescents are often discouraged by long boring assessments, assessments should be quick and motivating. We used the YouGrabber® system (YouRehab Ltd., Schlieren, Switzerland), a glove-based system with computer games that provides adults (e.g. [10]) and children ([11]) with brain lesions a motivating training. We investigated a particular game in which children had to steer an airplane through a cloud-free path by adequately applying elbow flexion or extension movements. We suggested that the relationship between the applied elbow movements and the ideal path of the airplane could be used as a measure reflecting the ability to selectively control elbow movements. We investigated the validity of this approach. We hypothesized that: (i) SVMC correlated positively (moderate size) with other clinical outcomes (e.g. muscle strength, trunk control), with higher scores reflecting better motor control. We expected the highest (strong) correlations with the expert opinion of an experienced occupational therapist who was instructed to rate the performance of the child during the game as physiological or compensatory. We expected negative fair to moderate correlations with clinical outcomes whose higher scores reflected poorer performance [e.g. spasticity as quantified with the modified Ashworth Scale (MAS) or manual ability with the Manual Ability Classification System (MACS)], (ii) compared to conventional clinical outcome measures, measures of SVMC could distinguish better between children who performed the game with physiological versus compensatory movements, and (iii) assuming that factors influencing physiological movement performance, such as spasticity or strength, might limit movement performance under more difficult (e.g. more accurate or faster) conditions, measures of SVMC became poorer when game difficulty was increased.

## Methods

### In- and exclusion criteria

In- or outpatients (convenience sampling) of the Rehabilitation Centre Affoltern am Albis (University Children’s Hospital Zurich, Switzerland) were recruited between August 2014 and August 2015. Included were participants aged 5–20 years, capable to understand and follow instructions, able to sit in an upright position for ≥ 45 min, with conditions affecting the central nervous system and resulting in impairments of the upper extremities, and ≥ grade 2 according to manual muscle testing of biceps brachii and ≥ 20° range of motion (ROM) against gravity of the elbow joints. Exclusion criteria were open wounds, severe spasticity in the elbow joint (MAS ≥ 4), athetosis, botulinum toxin injections or orthopedic interventions in the last 3 months. Furthermore, participants should not have visual problems for computer-assisted systems.

### Procedure

Participants were extensively characterized by various motor and cognitive assessments on body function and activity levels. All measurements occurred at the rehabilitation center in a quiet environment. The measurements lasted approximately one hour: 30 min for functional tests and 30 min for measurements on the YouGrabber® system. First, the clinical and functional assessments were performed. Active ROM, the MAS, and the Manual Muscle Test (MMT) were measured by a trained therapist and tested in this sequence so that joint limitations were known before measuring muscle force. The Trunk Control Measurement Scale (TCMS; performed by a qualified physiotherapist) and Test Of Non-verbal Intelligence (TONI-4; performed by a neuropsychologist) were performed during the same week. In some participants, the TONI-4 assessment had been performed several weeks in advance; these scores were used to reduce the stress for the participant. The MACS was routinely assessed for children with Cerebral Palsy (CP) and additionally provided by physicians to score children with other diagnoses.

### Assessments on body function domain

To score the spasticity with the MAS, the therapist moved the elbow first slowly through the full range of motion and then three times fast. Because spasticity is velocity-dependent, the therapist evaluated spasticity by judging changes in resistance [12]. Score 0 = no increase in muscle tone and 4 = affected part rigid in flexion or extension.

The MMT measures all the muscles which are involved in the tested movement and not an individual muscle [13]. It consists of a scoring scale from 0–5, where score 0 means paralyzed and a grade of 5 means “normal” muscle strength (i.e. full end-point range against maximal resistance). The therapist shows a movement passively, and if patients can move to its end range against gravity, a minimal score of 3 is achieved. To achieve a higher score, therapists give a manual resistance against contracting muscle groups. In this study, MMT of shoulder and elbow (shoulder flexion, abduction, and external rotation, elbow flexion and extension, and scapula elevation) were scored on a scale from 0 to 5 (maximal score 60). MMT scores for elbow flexion (performed if possible in supination) were notated separately. The MAS of the elbow for each arm was measured. The TCMS assesses trunk control in children with CP [14] The trunk plays a role as a stable base of support and an actively moving body segment. Three main components of trunk control (i.e. static sitting balance, selective movement control and dynamic reaching in sitting position) are measured during functional activities (i.e. movements of the upper and lower extremities). Static sitting balance includes 5 items consisting of sit upright and hold the position, lift the arms, cross the legs or abduct one leg. Selective movement control contains 7 items such as lean forward and backward, touch the table with the elbow, lift the pelvis, rotate upper/lower trunk and shuffle movement. Reach forward, backward and across the midline are the 3 items of the dynamic reaching condition. A total of 58 points can be achieved in the 15 items, indicating a good trunk control.

The TONI-4 is used in patients with limited linguistic or motor abilities [15]. Abstract reasoning and problem solving are the two components of intelligence that are measured. The test includes 60 items, where the first 19 items are for 6 to 9 years old children and the remaining 41 items are for the older ones. The items contain a sequence of abstract figures and one missing figure. Each item is scored with 1 for a correct answer or 0 point for an incorrect answer. Difficulty is ascending from first to the last item. Percentile scores between 25th and 75th percentile stand for an average age-standardized performance.

### Assessments on activity domain

The MACS is a practical observation-based classification system for manual ability in children with CP. It is scored by professionals and describes the children’s handling of objects in activities of daily life [16, 17]. The MACS has 5 levels: a child with level I handles objects easily and successfully whereas a child with level V does not handle objects and has severely limited ability to perform even simple actions.

### YouGrabber® measurements

**Fig. 1:**
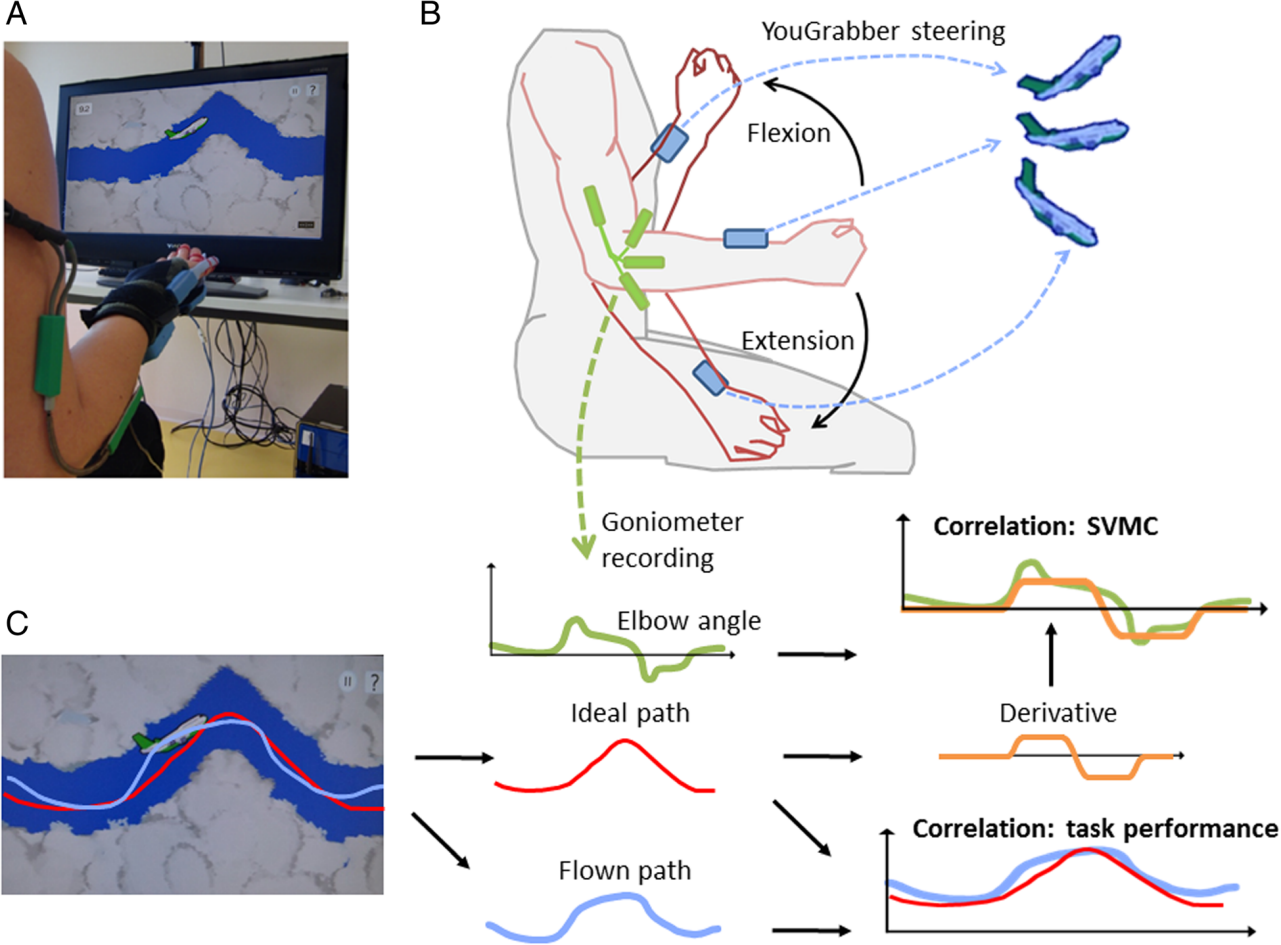
**a** Picture showing a participant playing the exergame. The goal for the child was to steer the airplane through the middle of the clouds-free path. Participants controlled the airplane by elbow flexion (airplane angle upward) and extension (airplane angle downward) movements. Electrical goniometer sensors, YouGrabber® data gloves with accelerometers and the airplane scenario are shown. **b** While the averaged accelerometer data of the left and right blue YouGrabber “boxes” were used to control the angle of the airplane, electrogoniometers recorded the actual elbow flexion and extension movements. **c** Besides the automatically provided game score (i.e. the percentage of time correctly on the path), we calculated various measures including the correlation between the ideal path and the airplane trajectory (as another measure reflecting task performance), and the correlation between the derivative of the ideal path and the elbow movements. This latter correlation served as our measure of selective voluntary motor control (SVMC)

The YouGrabber® system includes data gloves in different sizes, an infrared tracking camera, a large monitor with speakers and a personal computer (Fig. 1). The accelerometers on each glove allow the system to measure arm and hand movements. Tracking with the infrared camera permits measurements in space. Vibration motors attached to the gloves provide haptic feedback while the different game scenarios provide visual and auditory feedback. Various movements such as fine finger movements, reaching, grasping, elbow flexion and extension, arm lift and others can be trained. Depending on the game, individual movements can be selected. The system appears feasible in children with various diagnoses [11].

For this study, participants were equipped with customized YouGrabber®-gloves and worn them with supinated forearms. Participants sat in front of the monitor (Fig. 1a). Common sitting positions (e.g. wheelchair or wedge-shaped bolster) were used. After a short and standardized instruction, the therapist showed the movements by manually moving the arms of the child. We instructed the child to keep the airplane in the middle of the cloud-free path by performing bilateral arm movements: the airplane should be steered upwards with elbow flexion movements and downwards with elbow extension movements (Fig. 1b). If the airplane touched the clouds, the participants received a haptic feedback via the vibration motors, visual feedback via an orange colored airplane and auditory warning signals. The accelerometer signals of the right and left forearm were used to calculate the vertical angle of each forearm. This angle was directly related with the pitch angle of the airplane on the screen and, therefore, to the vertical speed of the plane. For example, a strong flexion of the elbow led to a steep pitch angle and a high upward speed of the airplane.

Despite that children were instructed to steer with isolated elbow flexion and extension movements, compensatory movements such as shoulder flexion or extension or changes in trunk position changed the angle of the forearm (with the accelerometers) and therewith the angle of the airplane. Therefore, we positioned DTS 2D electrical goniometer sensors (Biometrics LTD, Newport UK) with flexible axes and compatible with the Noraxon TeleMyo DTS System (Noraxon U.S.A Inc., Scottsdale AZ) over each elbow joint. The goniometers allowed us to measure angular changes of each elbow joint in two dimensions at 1000Hz. We recorded the data with simultaneous video recordings of the child and the game with MyoResearch XP Master Edition software (Velamed GmbH, Cologne, Germany) so that we could synchronize the output of the goniometers and the airplane game.

The children were familiarized with the game and played it at least once before the measurement started.

After the practice trial, measurements started with the basic airplane-game condition (broad path, 50 % speed). Game-difficulty was then increased, and the following conditions were performed in random order: condition speed (broad path, 80 % speed), condition path (narrow path, 50 % speed) and condition both (narrow path, 80 % speed). Finally, a control condition was performed at the end with the same settings as the first basic condition (broad path, speed 50 %) to investigate whether fatigue and/or learning effects might have influenced the scoring. Each condition lasted 90 s. We calibrated the system repeatedly according to the patients’ active range of motion of the elbows (i.e. at the beginning, before the block with the increased complexity and before the control condition). After each condition, there was a break of approximately 30 s. Here, the therapist repeated the instructions.

Compared to the commercially available airplane game, our airplane software was adapted for this study. We could program the flight path, so all children had to steer through the same trajectory and difficulty levels could be well compared. Besides receiving the regular game performance (i.e. the percentage of time on the cloud-free path [%]), we could now also record (at 33 Hz) the upper and lower boundary of the cloud-free path and the actually flown path of the airplane (Fig. 1a, c).

After each game, the therapist who supervised the measurements provided his/her expert opinion, whether the child had performed therapeutically desired physiological movements or whether compensatory movements were performed (see also [18]). We used this expert’s opinion because a recent review on psychometric properties of upper extremity tests in children showed that there are currently no psychometrically well-investigated outcome measures assessing SVMC [19]. We had no standardized procedure, but therapists overlooked the performance of the child and took into consideration whether elbow flexion and extension movements were performed isolated, i.e. not as part of synergistic patterns, and without accompanying movements from adjacent joints (i.e. wrists or shoulders) or the trunk. Due to the bilateral performance, therapists could not judge mirror movements (see methodological considerations section).

### Data analysis

The x and y coordinates of the upper and lower boundaries of the cloud-free path and the actually flown airplane path as functions of time were exported for further analyses with MATLAB (The MathWorks Inc., Natick, Massachusetts, USA). The x and y coordinates of the upper and lower boundaries were used to calculate the midline reflecting the ideal path. A Butterworth low pass filter (0.5Hz) was used to smoothen the trajectory due to small irregularities in this curve. Then, we performed correlation analyses between the trajectories of the airplane and the ideal path [r = corrcoef (x, y)] in MATLAB (Fig. 1c). This should reflect how well the child achieved the goal of flying at the midline of the cloud-free path.

As the forearm angle influenced the vertical speed of the airplane, we had to correlate the goniometer data (left and right arm separately) with the derivative of the ideal trajectory. This quantified how well the desired elbow movements corresponded with the actually performed elbow movements, so this became our measure quantifying SVMC (see also Fig. 1c). The derivative of the ideal path was used in the correlation analysis, as the arm angle reflects the vertical velocity of the airplane (i.e. the larger the angle, the steeper the airplane flew, Fig. 1b). Data for each arm and each condition were separately analysed. Data were grouped according to whether the arm was more of less affected (rather than left or right). These analyses required time to process. Therefore, the therapist was unaware of the outcomes of these measures when scoring the performance of the participant as physiological or compensatory, which reduced bias.

### Statistics

Statistical analyses were performed with SPSS 19.0 (SPSS Inc., Chicago, Illinois, USA).

Data were presented as mean ± standard deviation (SD) or median and inter-quartile range, depending on whether data were normally distributed or not (checked using the Shapiro-Wilk test).

Construct validity: Depending on whether data were normally distributed or not or whether outcomes were ordinal scaled Pearson’s (r) or Spearman (ρ) correlation coefficients were calculated. Point-biserial correlations were calculated between the dichotomous expert opinions of the occupational therapists (i.e. physiological versus compensatory movements) and various outcomes (similar to [18]). Correlation coefficients were interpreted as follow: 0.00–0.25 no to little; 0.25–0.50 fair degree of relationship; 0.50–0.75 moderate to good relationship; 0.75–1.00 very good to excellent relationship.

Discriminative validity: To determine whether the clinical measures, game score and new SVMC measures could differentiate well between participants who were categorized by the expert as performing physiological versus compensatory movements, we conducted Receiver Operating Characteristics (ROC) analyses. In short, the scores of each measure (i.e. clinical measure, game score or SVMC measure) were ranked together with the corresponding therapeutic scoring (i.e. physiological or compensatory). Then, the sensitivity [i.e. the proportion of positives (i.e. those performing compensatory movements) that are identified as such] and specificity [i.e. the proportion of negatives (i.e. those performing physiological movements) that are identified as such] were calculated between each successive score (i.e. clinical measure, game score or SVMC measure). The best cut-off value to distinguish between participants using compensatory versus physiological movements has the best combined sensitivity and specificity. Therefore, we determined the cut-off values with the highest Youden-Index (=Sensitivity + Specificity −1). The area under the curve (AUC) was presented as an indicator of the accuracy of the ROC-analysis. The AUC was considered acceptable (0.7–0.8), excellent (0.8–0.9) or outstanding (≥0.9) [20].

Differences: Differences between 2 independent groups were compared with a t-test or Mann-Whitney-U test [depending on measure scale (e.g. interval versus ordinal scale) or normal versus not normal distribution]. Differences between multiple game conditions were analysed with a repeated-measures ANOVA or Friedman’s test. Consecutive pair-wise testing was performed with paired-t-tests or Wilcoxon signed rank tests. A Bonferroni’s correction was applied for multiple comparisons. We used pairwise deletion of missing data.

## Results

We recruited 33 children and adolescents undergoing rehabilitation in our center. These participants were on average (± SD) 12.6 ± 3.6 years old (range 5.2–19.9 years; *n* = 33). Diagnoses and other clinical characteristics are presented in Table 1. In 3 children we could not detect upper limb impairments with our clinical test battery, despite their diagnoses (see also Table 1). The more affected hand was defined according to the diagnosis or, in children bilaterally affected, as the non-dominant hand. In one child only, the more affected hand was also the dominant hand. The total MMT score was on average 49.2 ± 9.1 from 60 points (range 26–60; *n* = 26). Elbow flexion MMT scores were lower for the more affected side (median = 4.0; IQR = 3.5–5.0) compared to the less affected side (median = 5.0; IQR = 3.8–5.0; *p* = 0.001). TCMS scores amounted to 36.2 ± 15.1 from 58 points (range 5–57; *n* = 29). TONI-4 percentage scores were on average 42.2 ± 30.8 % and varied between 2–96 % (*n* = 31). Due to upper limb limitations or reduced compliance, we could calculate an MMT total score only for 26 children. We could not obtain a TONI-4 score for 2 children (below the age of 6 years, i.e. too young to be tested with the TONI-4) and the TCMS was not obtained in 4 children (these children were not able to sit unsupported).

**Table 1:** Participants characteristics

Measure	Category	Number
Diagnoses	Cerebral Palsy	15
	Stroke	4
	Traumatic brain injury	3
	Encephalitis	2
	Others	9
Affected upper extremity^a^	Bilateral	12
	Left	13
	Right	5
Less affected side	Left	7
	Right	26
MACS	I	14
	II	12
	III	5
	IV	2
MAS^b^		
More affected side	0	19
	1	3
	2	4
	3	2
Less affected side	0	26
	1	1
	2	1

*Abbreviations*: *MACS* Manual Ability Classification System, *MAS* Modified Ashworth Scale

^a^In 3 children we could not detect upper limb impairments with our clinical test battery, despite their diagnoses

^b^Missing data: *n* = 5

### Game performance and measure of SVMC

**Fig. 2:**
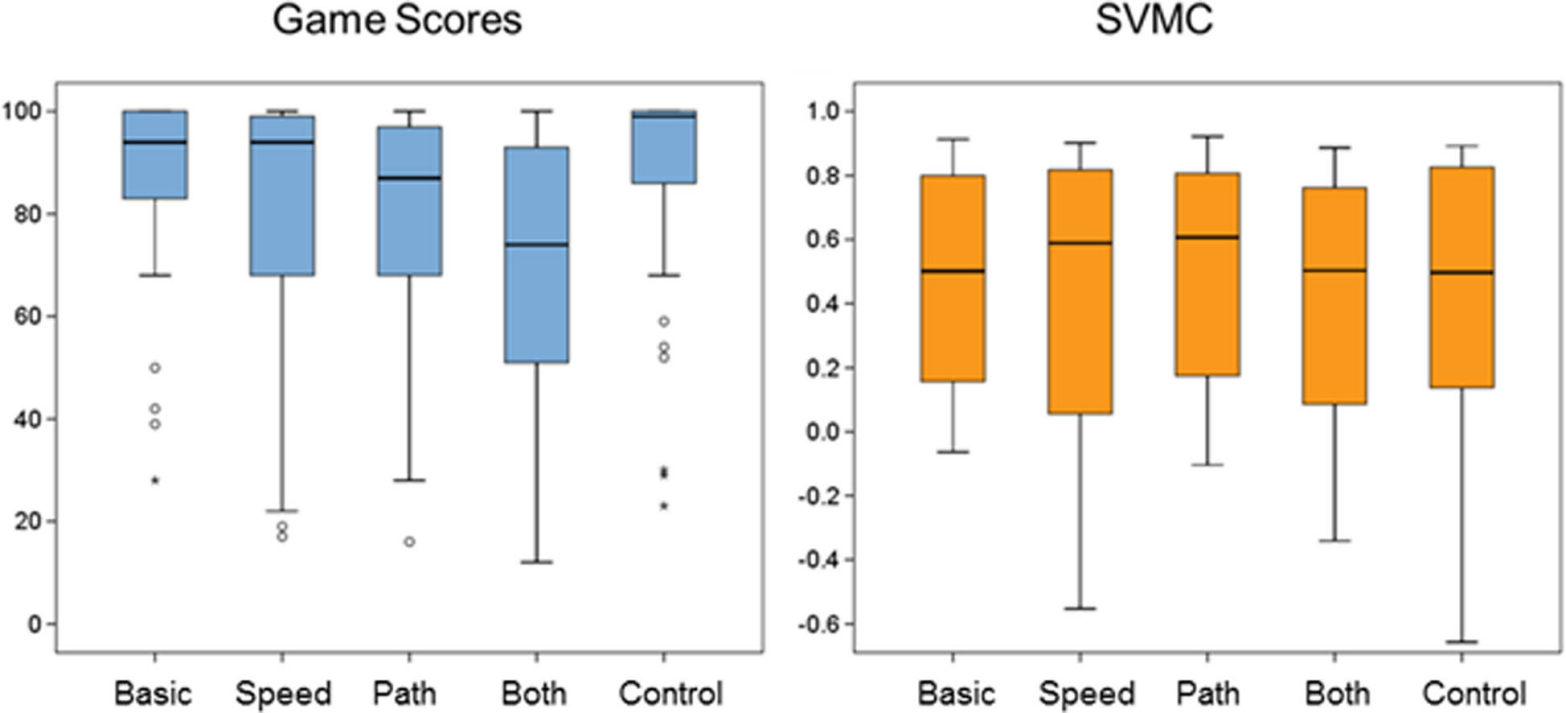
The performance of game scores and SVMC during the game conditions. Box-plots showing the game scores (i.e. the percentage of time correctly on the path), and our measure of selective voluntary motor control (SVMC; i.e. the correlation between the derivative of the ideal path and the elbow movements). While game scores decreased with increasing difficulty, SVMC scores remained similar

A Friedman’s test showed significant differences between the game performance scores (i.e. the percentage of time on the cloud-free path, Fig. 2, Additional file 1: Table S1) of different difficulty levels, i.e. different game conditions (*p* < 0.001). The participants achieved comparable game scores during the first basic condition and final control condition (*p* = 0.55, see Fig. 2). Compared to the game scores obtained during the basic condition, they scored significantly poorer during the speed (*p* = 0.003), path (*p* < 0.001), and both (*p* < 0.001) conditions. Game performance in the both condition was poorer compared to the path and speed condition (for both: *p* < 0.001). Finally, game performance in the speed condition slightly exceeded that of the path condition, but the difference (*p* = 0.039) was not significant after correction for multiple comparisons (α = 0.007).

The similarity between the actually flown path and the ideal path varied little between game conditions (mean correlation coefficients: 0.78 for the basic and 0.79 for the control condition, 0.73 for the speed and both conditions and 0.76 for the path condition). These coefficients also differed between the conditions (*p* < 0.001). However, pairwise comparisons showed only differences between the both condition versus the basic condition (*p* < 0.001), the control condition (*p* < 0.001), and the path condition (*p* = 0.01).

The SVMC measure varied widely among participants (Fig. 2 and Additional file 1: Table S1) and did not differ between the conditions (more affected arm: *p* = 0.78; less affected arm: *p* = 0.44) and between the more and less affected side (0.36 ≤ *p*-value ≤ 0.68). For each condition, the correlation between the ideal path and the actually flown path was significantly higher than the correlation coefficients reflecting our measure of SVMC (*p* < 0.001 for each condition).

Finally, the number of children scored by the occupational therapist who performed physiological movements did not differ between the conditions (basic: *n* = 16; speed: *n* = 16; path: *n* = 18; both: *n* = 16; control: *n* = 20; *p* = 0.33).

### Relationships between SVMC and other measures

As we found no significant differences in SVMC between the different conditions and more or less affected side, we presented the correlation analyses only for the SVMC of the more affected arm for the basic condition (Table 2). We found fair correlations between SVMC and age and elbow flexion muscle strength of the less affected arm and very good correlations between SVMC and the game score and SVMC and the correlation coefficient calculated between the ideal path and the actually flown path. The correlations with the other clinical variables were moderate to good (see Table 2). The directions of the correlations (i.e. positive or negative) were as expected.

**Table 2:** Relationships between selective voluntary motor control, expert opinion, and various measures

Measure		SVMC^a^	Expert opinion^b^	Number
Patient characteristics	Age	0.31 (0.08)	0.44 (0.01)	33
Clinical outcomes	MAS	−0.58 (0.001)	−0.61 (0.001)	28
	MMT Biceps MA	0.51 (0.002)	0.49 (0.004)	33
	MMT Biceps LA	0.33 (0.051)	0.39 (0.02)	33
	MMT Total	0.65 (<0.001)	0.58 (0.002)	26
	TONI-4	0.50 (0.004)	0.41 (0.02)	31
	TCMS	0.74 (<0.001)	0.70 (<0.001)	29
	MACS	−0.63 (<0.001)	−0.58 (<0.001)	33
Exergame scores and new SVMC measure	Game score	0.86 (<0.001)	0.56 (0.001)	33
	Flight/ideal	0.85 (<0.001)	0.63 (<0.001)	33
	SVMC MA		0.83 (<0.001)	33
	SVMC LA		0.82 (<0.001)	33

All correlations are presented for the basic condition. Correlations in the SVMC column were based on goniometer data obtained from the more affected side

*Abbreviations*: *MAS* Modified Ashworth Scale, *MMT* Manual Muscle Test, *MA* more affected side, *LA* less affected side, *TONI-4* Fourth version of the Test of Non-Verbal Intelligence, *TCMS* Trunk Control Measurement Scale, *MACS* Manual Ability Classification System, *Flight/ideal* correlation between the ideal path and the actual flown path, *SVMC* Selective Voluntary Motor Control, *n* number of participants included in the analyses

^a^Spearman correlation coefficients and ^b^point-biserial correlation coefficients with *p*-values between brackets

We also correlated the expert opinion of the occupational therapist with age, clinical outcomes and various game and goniometer derived measures (Table 2; we presented these correlations also for each separate condition in Additional file 2: Table S2). In general, the sizes of the correlation coefficients were comparable when correlating the SVMC measure or the expert’s opinion with age or the clinical measures. Correlations between game scores and the SVMC measure were higher than between game scores and the expert’s opinion. The correlation coefficients between the SVMC measure and the expert’s opinion (both measures should reflect SVMC) were among the highest.

**Fig. 3:**
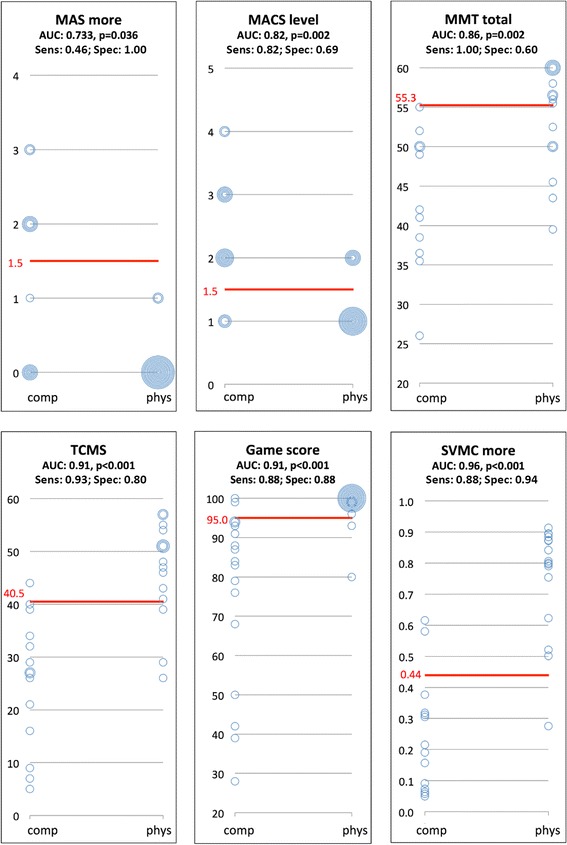
Receiver Operating Analyses showing sensitivity and specificity of various clinical, game performance and selectivity measures. The size of the bubble reflects the number of participants. Sensitivity is defined as the proportion of positives (i.e. those performing compensatory movements) correctly identified as such (e.g. for the SVMC measure 15/17 = 88 %). Specificity is defined as the proportion of negatives (i.e. those performing physiological movements) correctly identified as such (e.g. for the SVMC measure 15/16 = 94 %). Abbreviations: comp, compensatory; phys, physiological; MAS, Modified Ashworth Scale; more, more affected side; MACS, Manual Ability Classification System; MMT, manual Muscle Testing; TCMS, Trunk Control Measurement Scale; SVMC, Selective Voluntary Motor Control; AUC, Area Under the Curve; sens, sensitivity; spec, specificity

We used the expert opinion scores also to determine the ability (i.e. sensitivity and specificity) of the various measures to distinguish between participants performing selective voluntary physiological versus those performing compensatory movements while playing the airplane game. Using ROC analyses, we analyzed the ability of clinical and game scores, including the SVMC measure, to discriminate between these participant groups (shown only for the basic condition in Fig. 3). The AUC’s of the ROC analyses were outstanding for the TCMS, game score and the SVMC measure (Fig. 3). MAS scores and elbow flexion MMT scores (both Youden Index of 0.46, latter not shown) and total MMT scores of the more affected side (Youden Index 0.60) showed much smaller sensitivity and specificity than game performance (Youden Index was 0.76 for the % on the correct path as well as for the similarity between the ideal and actually flown trajectories, latter not shown) or trunk control (Youden Index was 0.73 for TCMS scores). The highest sensitivity and specificity for discriminating between compensatory and physiological game performance was found for the SVMC (Youden Index 0.82). The results for the other game conditions were quite comparable and therefore not shown.

## Discussion

In this study, we investigated whether a specific game in combination with electrogoniometer measurements could provide a measure reflecting SVMC in children with motor disorders of the central nervous system. We named this study “a pilot study” because originally it was not our purpose to construct a new measure reflecting SVMC. Rather we discovered during a project investigating the prerequisites participants needed to fulfil to train with this exergame that our instructions to the children who were playing the game, namely to perform isolated elbow flexion and extension movements to steer the airplane, fitted rather well (but not perfect, see methodological considerations) to the definition of SVMC, as proposed by Sanger et al. [5].

The main results were the following: (i) our novel SVMC measure correlated moderately with various upper limb capacity measures and excellently (and better than most other clinical measures) with the opinion of experienced occupational therapists indicating construct validity. (ii) The sensitivity and specificity of the SVMC measure differentiating between participants who perform physiological versus compensatory movements were superior compared to other clinical measures and game scores, indicating discriminative validity. (iii) While game scores differed significantly between game conditions of different difficulty grades, the measure of SVMC did not differ between these conditions.

### Correlations with clinical outcomes and exergame scores

We could accept our first hypothesis, as our SVMC measure correlated moderately to good with other clinical outcomes reflecting motor control (e.g. muscle strength or trunk control). From the clinical outcomes, the TCMS correlated best with SVMC. The TCMS is a valid and reliable tool assessing static sitting balance and dynamic sitting balance, whereas the latter is divided into two subscales, “selective movement control” and “dynamic reaching” [14, 21]. We expect that the high correlation between the TCMS and our SVMC measure can partly be explained by the fact that selective upper extremity movements require a stable base of support, i.e. a stable trunk. In addition, both measures assess (partly) selective voluntary movements (of trunk or arms). As the topographical localization of arms and trunk in the sensorimotor cortex are relatively close to each other, a brain lesion might, therefore, result in similarly affected TCMS and SVMC upper limb scores.

Interestingly, a recent study presented correlations between the Selective Control Assessment of the Lower Extremity (SCALE) and clinical outcomes. The relationship between the SCALE and leg MMT scores amounted to 0.88 [22]. This is a stronger relationship than we found when comparing our SVMC measure with upper extremity MMT scores. We assume that such a difference could be caused by the different SVMC measures (i.e. ordinal-scaled SCALE versus our interval-scaled SVMC measure). Another explanation could be the more differentiated selectivity of the arms compared to the legs. Strength and selectivity share a common neurophysiological basis (e.g. the sensorimotor cortex, pre- and supplementary motor area, corticospinal tract), and we require both strength and selectivity to perform physiological movements. However, the reduced differentiated selectivity of the legs compared to the arms might result in a stronger relationship between SVMC and strength of the legs compared to SVMC and strength of the arms.

Spasticity could affect selectivity in a negative way. Indeed, the MAS and our SVMC measure correlated moderately (ρ = −0.58) and well comparable to the relationship between the MAS and the SCALE (ρ = −0.55, see Balzer et al. 2015). In our study, however, the MAS was not a good classifier to distinguish between participants performing selective versus compensatory movements.

Interestingly, most correlations between SVMC and game scores were higher than correlations between SVMC and the clinical scores. This could indicate that children who achieve high game scores are more likely to play their exergame using physiological movements. Perhaps specific exergame scores could prove, in the near future, useful to document changes in selective movement performance.

### Expert opinion

As a review by Gerber et al. (2016) [19] showed that there are currently no psychometrically well-investigated upper extremity tools assessing SVMC in pediatrics, we used the expert opinion as a comparator. We expected and indeed found higher correlations between the expert opinion of an experienced occupational therapist and our SVMC measure compared to most other measures. Also, the ROC analyses showed that the SVMC measure had the highest combined sensitivity and specificity compared to the other measures. Therefore, we could accept the second hypothesis and assume that our new measure indeed reflects physiological selective movements. The opposite reasoning is also of interest. In clinical practice, therapists continuously evaluate the quality of movement performance. These findings show that experienced therapists are well able to distinguish between compensatory and physiological movements. For clinical evaluation and decision-making, a therapist’s opinion seems both pragmatic and valid in categorizing young patients. As our sample was rather heterogeneous concerning diagnoses, severity, and age, these findings appear relatively robust, i.e. the generalizability seems large.

We could not accept the third hypothesis because the measures of SVMC did not become poorer with increasing game difficulty. While we assumed that factors such as spasticity or reduced strength might limit movement performance under more difficult (e.g. accurate or faster) conditions, this did not occur. In addition, the number of patients who were scored as physiological remained relatively constant during the different game conditions. On the one hand, this might indicate that SVMC does not become largely influenced by the difficulty of the task; participants performed physiological movements, no matter if conditions were easier or more difficult. On the other hand, perhaps the difficulty of the task was still not challenging enough for provoking compensatory movements. Although game scores deteriorated during more difficult conditions, many participants were still able to play the exergame well. This issue needs to be further investigated.

### Methodological considerations

Despite that this novel SVMC measure appears to be a valid approach to quantify SVMC, we propose the following changes to improve the protocol:

(i) Participants should perform unilateral (and not bilateral) isolated joint movements to be able to assess mirror movements. Mirror movements can occur in patients with ipsilateral brain reorganization, i.e. where both hands receive corticospinal projections from one hemisphere (for an excellent overview see [23]). Patients who acquired the lesion early during development frequently show a useful grasp function with their paretic hand or even preserved individual finger movements (e.g. [24]). Brain damage acquired around term birth or postnatally shows mostly no useful hand function, despite the presence of ipsilateral tracts. However, all patients controlling both the paretic and non-paretic hand with the contralesional hemisphere show during voluntary one-handed movements involuntary ‘mirror movements’ of the contralateral hand. Such mirror movements are a sign of reduced SVMC (referring to not performing “isolated activations” in the definition of SVMC) and need therefore to be monitored rather than included in the target movement.
(ii) The protocol needs additional monitoring of adjacent joints and trunk when performing an isolated movement, to quantify objectively co-activations (indicating reduced SVMC).
(iii) The current quantification of calculating correlation coefficients between the goniometer trajectories and the derivative of the ideal trajectory is not ideal. Preferably, a root mean square error (RMSE) between the target trajectory and joint movement should be calculated. For this, the control and calibration of the game need to be changed, i.e. the game should become position controlled.
(iv) Participants were instructed to steer in the middle of the cloud free path. In a new game, the exact trajectory should be displayed on the monitor to improve the clarity of the task to the participant. Currently, we are implementing these changes and hypothesize that the new protocol will become even more specific and sensitive in quantifying SVMC.
(v) Recently, Wagner et al. [25] published the Selective Control of the Upper Extremity Scale (SCUES), which is a new clinical tool assessing SVMC of the upper extremity. The authors determined the content validity and the intra- and inter-rater reliability of the SCUES. First results showed that the SCUES was reliable (ICC values exceeded 0.75, except for the inter-rater reliability of the shoulder where the ICC was 0.72). Furthermore, the SCUES correlated significantly with the Shriners Hospitals Upper Extremity Evaluation (ρ = 0.69), but no significant correlations were found with the box and block test or the MACS [25]. We are currently translating the SCUES in the German language according to recommended guidelines [26, 27] and will investigate its psychometric properties after the translational process is finished. We will then use the SCUES as a comparator to validate our new playful SVMC measure.
(vi) We will expand the measurements to additional upper and lower extremity joints. For the lower extremity, we will validate the approach with the SCALE, which is a tool assessing SVMC of lower extremity joints [28]. The German version of this tool has already been published [22]. It proved to be a reliable (intra- and inter-rater ICCs exceeding 0.9) and a valid clinical tool to assess SVMC of the legs in children with spastic CP.
(vii) Finally, reference values obtained in healthy children and adolescents might be needed to determine age-dependent changes in SVMC.

## Conclusions

We showed in this pilot study that we developed an objective and sensitive measure quantifying SVMC. Importantly, we could determine SVMC while the participants were playing an exergame, i.e. participants remained motivated to perform the assessment. We proposed several improvements to our protocol and are currently working on realizing this new setup. Nevertheless, as the current results are promising, this study contributes to the existing literature on SVMC assessments. The need for such assessments is substantial, as the question of whether rehabilitation specialists should focus on stimulating recovery or promoting improved function and independence by allowing compensatory movement strategies will remain a matter of debate in the coming years.

## Additional files

Additional file 1: Table S1. Performance and ROC analyses of game scores and SVMC. (DOC 29 kb)
Additional file 2: Table S2. Relationships between expert opinion and various measures for each condition. (DOC 35 kb)

## Abbreviations

AUC: Area under the curve
comp: Compensatory
CP: Cerebral palsy
ICF: International classification of functioning, disability and health
MACS: Manual Ability Classification System
MAS: Modified Ashworth Scale
MMT: Manual muscle testing
more: More affected side
phys: Physiological
ROC: Receiver operating characteristics
SCALE: Selective control assessment of the lower extremity
SCUES: Selective control of the upper extremity scale
sens: Sensitivity
spec: Specificity
SVMC: Selective voluntary motor control
TCMS: Trunk control measurement scale
TONI-4: 4^th^ edition of the test of non-verbal intelligence
