# Supplementary material for: Quantifying selective elbow movements during an exergame in children with neurological disorders: a pilot study
Source: J Neuroeng Rehabil. 2016 Oct 21;13:93. doi: 10.1186/s12984-016-0200-3 (PMC5073824; doi:10.1186/s12984-016-0200-3)
Supplement: Additional file 1: Table S1. — Performance and ROC analyses of game scores and SVMC. (DOC 29 kb) [file 12984_2016_200_MOESM1_ESM.doc]

Additional file 1: Table S1. Performance and ROC analyses of game scores and SVMC.

| Condition | Game scores (% on path) | | | | Gonio MA - ideal path | | | | Physiological  movements |
| --- | --- | --- | --- | --- | --- | --- | --- | --- | --- |
|  | Mean (range) | Cut-off | Sensitivity | Specificity | Mean (range) | Cut-off | Sensitivity | Specificity |
| Basic | 86.7 (28-100) | 95.0 | 0.88 | 0.88 | 0.46 (-0.06-0.91) | 0.44 | 0.88 | 0.94 | 16 |
| Speed | 80.5 (17-100) | 88.5 | 0.77 | 1.00 | 0.44 (-0.55-0.90) | 0.53 | 0.88 | 0.94 | 16 |
| Path | 77.9 (16-100) | 88.5 | 0.93 | 0.83 | 0.46 (-0.10-0.92) | 0.40 | 0.87 | 0.89 | 18 |
| Both | 69.2 (12-100) | 67.5 | 0.71 | 0.94 | 0.44 (-0.34-0.89) | 0.51 | 0.94 | 0.88 | 16 |
| Control | 85.8 (23-100) | 96.5 | 0.69 | 0.70 | 0.46 (-0.66-0.89) | 0.53 | 0.92 | 0.75 | 20 |

Please note game scores were expressed as % correct on path (i.e. the airplane flew between the clouds). The Selective Voluntary Motor Control (SVMC) measure reflected the correlation between the goniometer data of the more affected (MA) arm and the derivative of the ideal trajectory.
