# Supplementary material for: Quantifying selective elbow movements during an exergame in children with neurological disorders: a pilot study
Source: J Neuroeng Rehabil. 2016 Oct 21;13:93. doi: 10.1186/s12984-016-0200-3 (PMC5073824; doi:10.1186/s12984-016-0200-3)
Supplement: Additional file 2: Table S2. — Relationships between expert opinion and various measures for each condition. (DOC 35 kb) [file 12984_2016_200_MOESM2_ESM.doc]

Additional file 2: Table S2. Relationships between expert opinion and various measures for each condition.

|  | Outcomes | N | Basic | Speed | Path | Both |
| --- | --- | --- | --- | --- | --- | --- |
| Patient characteristics | Age | 33 | 0.44 (0.01) | 0.33 (0.064) | 0.32 (0.068) | 0.48 (0.005) |
| Clinical outcomes | MAS | 28 | -0.61 (0.001) | -0.60 (0.001) | -0.50 (0.007) | -0.52 (0.005) |
|  | MMT Biceps MA | 33 | 0.49 (0.004) | 0.28 (0.11) | 0.57 (0.001) | 0.56 (0.001) |
|  | MMT Biceps LA | 33 | 0.39 (0.02) | 0.18 (0.31) | 0.46 (0.007) | 0.39 (0.02) |
|  | MMT Total | 26 | 0.58 (0.002) | 0.42 (0.02) | 0.49 (0.01) | 0.48 (0.01) |
|  | TONI-4 | 31 | 0.41 (0.02) | 0.42 (0.02) | 0.53 (0.002) | 0.42 (0.02) |
|  | TCMS | 29 | 0.70 (<0.001) | 0.61 (<0.001) | 0.65 (<0.001) | 0.37 (0.052) |
|  | MACS | 33 | -0.58 (<0.001) | -0.45 (0.009) | -0.63 (<0.001) | -0.31 (0.078) |
| Game score | Game score | 33 | 0.56 (0.001) | 0.62 (<0.001) | 0.76 (<0.001) | 0.53 (0.002) |
|  | Flight/ideal | 33 | 0.63 (<0.001) | 0.65 (<0.001) | 0.58 (<0.001) | 0.40 (0.02) |
|  | SVMC MA | 33 | 0.83 (<0.001) | 0.59 (<0.001) | 0.77 (<0.001) | 0.70 (<0.001) |
|  | SVMC LA | 33 | 0.82 (<0.001) | 0.78 (<0.001) | 0.70 (<0.001) | 0.67 (<0.001) |

Presented are point-biserial correlation coefficients and, between brackets, p-values. Abbreviations: N, number; MACS, Manual Ability Classification System; MMT, Manual Muscle Test; MA, more affected; LA, less affected; TONI-4, 4^th^ version of the Test Of Non-verbal Intelligence; TCMS, Trunk Control Measurement Scale; Flight/ideal, correlation between the ideal path and the actual flown path; SVMC, Selective Voluntary Motor Control
